# Listening to two-talker conversations in quiet settings: the role of listeners’ cognitive processing capabilities for memory and listening effort

**DOI:** 10.1038/s41598-024-74085-1

**Published:** 2024-10-01

**Authors:** Chinthusa Mohanathasan, Janina Fels, Sabine J. Schlittmeier

**Affiliations:** 1 Work and Engineering Psychology, RWTH Aachen University, 52066 Aachen, Germany; 2 Institute for Hearing Technology and Acoustics, RWTH Aachen University, 52074 Aachen, Germany

**Keywords:** Psychology and behaviour, Health sciences

## Abstract

Listening to conversing talkers in quiet environments and remembering the content is a common activity. However, research on the cognitive demands involved is limited. This study investigates the relevance of individuals’ cognitive functions for listeners’ memory of two-talker conversations and their listening effort in quiet listening settings. A dual-task paradigm was employed to explore memory of conversational content and listening effort while analyzing the role of participants’ (*n* = 29) working memory capacity (measured through the operation span task), attention (Frankfurt attention inventory 2), and information-processing speed (trail making test). In the primary task, participants listened to a conversation between a male and female talker and answered content-related questions. The two talkers’ audio signals were presented through headphones, either spatially separated (+ /– 60°) or co-located (0°). Participants concurrently performed a vibrotactile pattern recognition task as a secondary task to measure listening effort. Results indicated that attention and processing speed were related to memory of conversational content and that all three cognitive functions were related to listening effort. Memory performance and listening effort were similar for spatially separated and co-located talkers when considering the psychometric measures. This research offers valuable insights into cognitive processes during two-talker conversations in quiet settings.

**Supplementary Information:**

The online version contains supplementary material available at 10.1038/s41598-024-74085-1.

## Introduction

Face-to-face conversations between two or more individuals are likely the most common mode of verbal communication. Multi-talker conversations occur in various settings, such as offices or schools, where one person acts primarily as a listener. This listener needs to remember what is said by the talkers to join the conversation later as a talker themselves or remember the content for later use. Thus, from a cognitive-psychological perspective, the listener’s task involves attentive listening, cognitive processing, and memorization of the conversational content. This task is demanding, even in quiet settings with perfectly comprehensible audio signals. The challenge arises from the strictly sequential and non-repeatable nature of speech signals presented at a specific speed^1^. The ease with which a person can listen to and cognitively process the heard speech and its content depends on the listener’s cognitive capacities and capabilities, such as working memory capacity, attention, and information-processing speed^2^. Furthermore, even in quiet settings where speech signals in conversations are perfectly intelligible, the listening effort of listeners to a two-talker conversation has been shown to vary with the availability of talker-related auditory spatial cues^3^. This study explores how listeners’ working memory capacity, attention, and information-processing speed influence listening effort and memory for conversational content when listening to two-talker conversations in quiet settings.

Listening effort refers to the amount of processing resources that a listener allocates to an auditory task when they aim for high-level performance or when the environment hinders speech understanding^4,5^. While memory performance can be measured by asking content-related questions, listening effort can hardly be measured directly. It is feasible to attain comparable performance results in the task associated with running speech in different listening conditions, while the degree of listening effort required for achieving such performance may vary between these conditions^3^. For this reason, a dual-task paradigm where two tasks are simultaneously performed is often utilized for measuring cognitive spare capacity as an indicator of listening effort^3,6^. The fundamental concept of the dual-task paradigm is that the cognitive resources available to a person are restricted^7^. Therefore, if one task (the primary task) is more resource-demanding, then fewer resources will be available for the second, parallel task (the secondary task). In listening research, the primary task is generally identified as the one that directly measures speech performance, such as speech recognition or, in our case, remembering conversational content. The secondary task is identified as the one additional task used to measure cognitive spare capacity, which is then interpreted as an indicator of listening effort. For example, if primary task performance is alike in two experimental conditions, A and B, observing reduced secondary task performance in condition A is interpreted as indicating greater listening effort in this experimental condition^4,5^. However, other outcomes can also be indicators of listening effort (see Ref.^8^).

The ease of language understanding model (ELU model) provides a theoretical framework by connecting auditory-perceptive information, language comprehension, and listening effort^2,3^. Although the ELU model was created to comprehend listening in adverse listening situations, it has the potential to elucidate the function of auditory-perceptive cues in memory processes during quiet listening situations^3^. Briefly, the ELU model distinguishes between implicit and explicit cognitive processing paths for understanding spoken language. If there is a discrepancy between the mental representation of a reduced or distorted auditory signal and long-term memory representations, the implicit route might not work, leading to the activation of the explicit route. Compared to the automated implicit route, the explicit route is slower and requires more resources. Consequently, the more explicit processing is needed, the more listening effort a listener needs for speech processing, understanding, and remembering what has been said. Here, individual working memory capacity becomes important. Working memory is the ability of a person to process and store information at the same time^9,10^. The greater a listener’s working memory capacity, the more processing resources are available to process, understand, and remember speech. These individuals can use different types of information and strategies to extract meaning from a message^2^. Often, and especially under adverse listening conditions, such as when competing speech maskers are in play^11^, individual working memory capacity appears to play a decisive role in speech perception and understanding^12,13^.

Individual differences in working memory capacity are arguably associated with the ability to focus attention and avoid distraction to meet demanding task goals^2,14,15^. Individuals with high working memory capacity demonstrate enhanced attentional modulation, allowing them to effectively allocate attentional resources. This heightened ability to manage attention may be particularly advantageous in effortful or divided attention tasks^2,16^.

Working memory is not only linked to the ability to control attention^17^, but also to the efficiency of information processing^15,18^. Information-processing speed is a cognitive function that assesses how rapidly individuals complete cognitive tasks^19^ and is essential for speech understanding to be successful and immediate^2,20^. For example, Homman et al.^18^ showed that high processing speed is necessary to allow working memory capacities to compensate for adverse listening conditions. Therefore, when investigating listening-related cognitive performance, particularly in the context of running speech, it is important to consider, measure, and account for individual working memory capacity, attention, and information-processing speed^2^. To the best of our knowledge, the present study is the first to accomplish this.

Considerable research has been directed towards investigating individual differences in speech understanding under noisy listening conditions, as evidenced by meta-analyses exploring the relationship between cognitive performance and speech-in-noise performance^21,22^. For example, Desjardins^23^ investigated the effects of working memory capacity, attention, and information-processing speed on listening effort and speech recognition performance in a dual-task paradigm under three background noise masker conditions (two-talker, six-talker, and speech-shaped noise). In all three background noise masker conditions, she found that speech recognition performance was correlated with working memory and processing speed, but it was not correlated with attention. Furthermore, in two of these masker conditions, listening effort was related to working memory capacity and processing speed. These findings suggest that individuals with greater working memory capacity and faster processing speed require less listening effort and show better speech recognition performance in background noise.

Although such studies provided valuable insights, for example, into how speech understanding depends on cognitive functions, they used mostly basic stimuli, such as single words or isolated sentences, and did not explore the intricacies of running speech. Speech understanding is a complex process that involves not only perceiving and identifying individual speech sounds and words but also integrating successively heard words, phrases, and sentences for participants to arrive at coherent and accurate representations of the conversational content^23^. In addition, previous investigations have examined listening abilities in adverse listening environments, such as noisy environments or when there are multiple talkers present who are not relevant to the task^24,25^. The everyday situation, in which an individual wants or needs to listen to an alternating conversation without background noise and memorize the conversational content while accounting for cognitive functions, has only been scarcely researched.

As far as we know, the only study that investigated such a situation was presented by Fintor et al.^3^. Here, the relevance of spatial separation (± 60°) versus co-location (0°) of two conversing talkers for a listener’s memory and listening effort in quiet listening environments was examined in two dual-task experiments. The participants were asked content-related questions right after listening to a conversation between two talkers to assess their memory of running speech content. In Experiment 1, a relatively simple number-judgement task was assessed as a visual secondary task, and in Experiment 2, this number-judgement task was combined with a visual letter-judgement task. The latter resulted in a switching task as a more demanding secondary task. Although memory performance was similar regardless of whether the two talkers’ audio signals were co-located or spatially separated, a performance benefit in the secondary tasks was observed in the latter condition. This suggests that the spatial separation of talkers was less demanding on cognitive resources than the co-location of the two talkers’ audio signals. Most importantly, Fintor et al.^3^ demonstrated that variations in listening effort can be measured in quiet listening environments without any background noise or task-irrelevant talkers. This study suggests that the concept of listening effort can be extended to scenarios that are typically viewed as favorable for listening performance.

While the study by Fintor et al.^3^ comes closer to everyday listening situations, it did not consider the listener’s cognitive functions, such as working memory capacity, attention, and information-processing speed. These cognitive functions are known to play a role in speech processing^2,12,16,26–28^ and may become more crucial when listeners are presented with running speech. Further research is needed to gain a more profound understanding of the cognitive functions involved in listening to running speech and their relationship with memory performance and listening effort in conversations involving two talkers, in quiet settings.

### Research intent

In the present study, we aimed to explore the relationship between listeners’ cognitive functions and the spatial separation versus co-location of two conversing talkers during a listening task and its impact on listening effort in quiet environments. To this end, we psychometrically measured three cognitive functions: working memory capacity^29^, attention^30^, and information-processing speed^31^. To assess memory and listening performance, a dual-task paradigm was used^8^. The primary listening task consisted of running speech and corresponding questions and was presented with recordings representing two co-located talkers (0°) or two spatially separated talkers (+ /– 60°). The secondary task was a vibrotactile pattern recognition task.

In line with Fintor et al.^3^, we argue that the spatial separation of talkers facilitates speech comprehension by activating the automated implicit route. This is because the auditory system likely relies on spatial location to discern perceptual objects, such as two talkers, as distinct entities. When talkers are co-located, more explicit processing is necessary to differentiate them. Additionally, the presence of two co-located talkers may be perceived as a deviation in the cognitive system, further necessitating explicit processing to resolve this discrepancy. Importantly, individual cognitive functions may influence the degree to which auditory spatial cues impact these processes.

## Methods

### Participants

Of the 30 participants who initially took part in the experiment, one had to be excluded due to impaired hearing, as determined by pulse pure tone ascending audiometry (AURITEC ear3.0 audiometer and Sennheiser HAD 280 headphones). The final sample included 29 participants (24 female, 5 male, 0 non-binary) aged between 18 and 37 years (*M* = 23.1 years, *SD* = 4.1) who reported normal or corrected-to-normal vision. These participants had normal hearing (< 20 dB hearing level in the frequency range between 250 and 4000 Hz) as assessed via audiometry. The participants were recruited through advertisements and were compensated with course credits or €15 for their participation.

### Ethics statement

Participants provided informed consent before participating. The research adhered to all the relevant national regulations, institutional policies, and by the tenets of the Helsinki Declaration, except for the experiments not being preregistered. The local ethics committee at the Philosophical Faculty of the RWTH Aachen University pre-approved a series of experiments to which the present experiment belongs (“Listening to, and remembering conversations between two talkers: Cognitive research using embodied conversational agents in audiovisual virtual environments”, 2021_08_FB7_RWTH AACHEN)*.*

### Stimuli, instruments, and apparatus

The experiment was developed in PsychoPy 2021.2.3 (Python 3.6.6^32^) and conducted on a Dell Latitude 3590 laptop. The laptop’s non-glare 15-inch screen was used to present all visual content. All auditory stimuli were played back using a Focusrite Scarlett 2i2 2nd Gen external sound card and Sennheiser HD 650 headphones. The auditory stimuli were taken from the AuViST database^33^. They consist of German sentences^34^ spoken by a professional female talker and a professional male talker. The auditory stimuli were binaurally presented at a distance of 2.5 m from the listener, either at the same position in space (0°; co-located condition) or at +/– 60° (spatially separated condition). The spatial auditory stimuli were created by convoluting the raw auditory stimulus with a head-related transfer function of the IHTA artificial head^35^. The sound pressure level was calibrated to 60 dB(A) at the ear canal entrance of the IHTA artificial head. For the two paper and pencil tests, the Renkforce RWF-SW-130 digital stopwatch was used to record time.

### Primary listening task

The heard text recall (HTR^3,8,34^) served as the primary listening task. The auditory stimuli were spoken coherent texts, each describing three generations of a family (grandparents, parents, and children), considering different aspects such as the family members’ age, professions, hobbies, and relationships with one another. Each text consisted of ten sentences (*M* = 42.0 s, *SD* = 4.1) and was presented as a conversation between one talker with a female voice and another talker with a male voice. Within one text, the turn-taking between the female and the male talkers occurred three to five times and was intended to simulate a natural conversation. Therefore, sentences closely related in content were spoken by the same conversational partner.

For each text, nine corresponding questions had to be answered. The questions asked for names of family members, relations between family members, and further information (e.g. profession). Each question remained until a reply was given. By pressing space and return, participants could skip a question, but they were unable to go back and answer a missed question or change their answer once they had already entered it. Each question could be answered with one to two words. The responses of the participants were manually scored afterward.

**Fig. 1:**
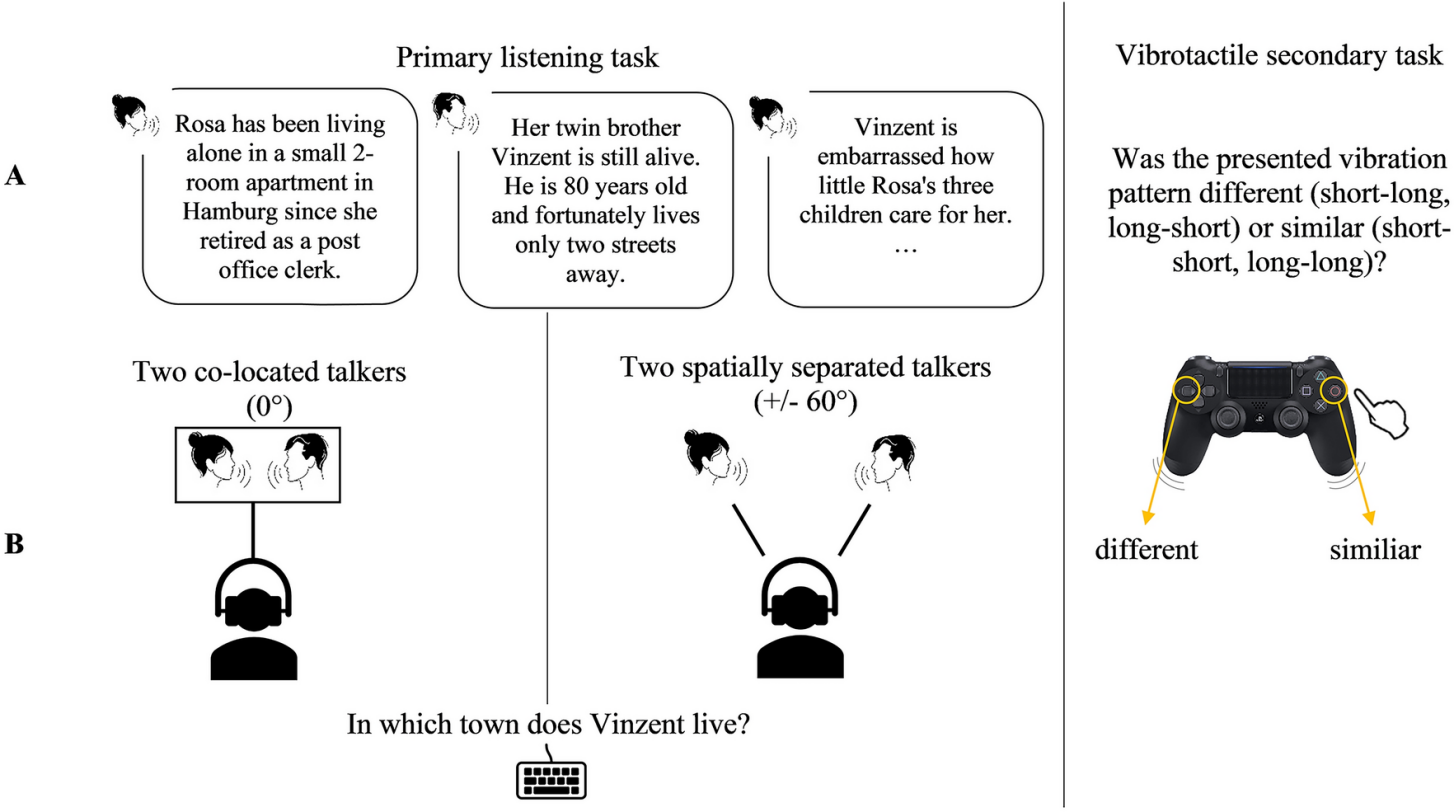
Illustration of the primary listening task and the vibrotactile secondary task. Left side: Illustration of the primary listening task, which was presented at 60 dB(A). (**A**) Excerpt of a two-talker conversation translated into English. (**B**) Illustration of the experimental design: Talkers’ audio condition was varied as a within-subject factor, with the audio of two talkers presented either at 0° (co-located condition) or at + /– 60° degrees (spatially separated condition). Right side: Illustration of the vibrotactile secondary task**.** In the vibrotactile secondary task, two similiar (short-short or long-long) or two different (short-long or long-short) vibration patterns were presented through a game controller.

Sample sentences and a sample question translated into English are shown in Fig. 1. For further information about and access to the stimuli and audio recordings, see Ref.^34^ and the AuViST database^33^.

### Vibrotactile secondary task

The vibrotactile pattern recognition task^6,8^ served as a secondary task. In this task, participants held a Sony Dualshock 4 Controller in their hands as it vibrated in four different ways: short-short, long-long, short-long, and long-short. Participants had to identify whether the presented vibrotactile pattern was the same (i.e. long-long) or different (i.e. long-short) by pressing the circle or the left arrow button, respectively, on the game controller (see Fig. 1). For each text, the number of tactile patterns is determined by the length of the sound files. In this task, a constant interstimulus interval of 1.5 s is given.

### Working memory capacity

The automated version of the operation span task (OSPAN^36^) was administered to measure participants’ working memory capacity. The automated OSPAN (aOSPAN^29^) is controlled by the participants using a mouse. Participants are presented with a visual sequence of consonants (F, H, J, K, L, N, P, Q, R, S, T, and Y) ranging from 3 to 7 letters that need to be recalled at the end. The consonant remained on the screen for 800 ms. Each consonant in the sequence is preceded by a math problem (e.g. “3/3 + 2 = ?”), followed by a proposed solution (e.g. “9”), and participants have to decide whether the proposed solution is “correct” or “incorrect” by clicking on a button. Letter recall is tested by asking participants to select consonants from a provided 4×3 consonant matrix. Participants were able to leave a consonant out by pressing “blank”, and they could correct the consonant they just entered by pressing “delete”. Altogether, three sets of each set size, ranging from three to seven (e.g. set size three equals three math operations and three consonants), were presented in random order, making for a total of 75 letters and 75 math problems.

The traditional absolute scoring method was used as a measure. This was the sum of all perfectly recalled sets. So, for example, if an individual correctly recalled 3 letters in a set size of 3, 4 letters in a set size of 4, and 3 letters in a set size of 5, his or her aOSPAN score would be 7 (3 + 4 + 0).

### Attention

Attention was measured with the Frankfurt Attention Inventory 2 (FAIR-2^30^). This psychometric test requires discriminating between target items and non-target items as quickly as possible without error. The FAIR-2 comprises two test pages, each containing 16 rows of 20 stimuli. The stimuli consist of shapes (circles or squares) with two or three dots placed inside. Targets in Version A are circles with three dots and squares with two dots, while distractors are circles with two dots and squares with three dots. When undertaking the FAIR-2 assessment, individuals are expected to draw a continuous line beneath each stimulus row and to designate every target with an upward spike through it. Participants have a three-minute time limit per test page.

In the FAIR-2, there are three indices: L, Q, and K. The L variable, derived from the German word “Leistung” (meaning “performance”), represents the sum of hits and indicates the number of correctly detected targets. The Q variable, derived from the German word “Qualität” (meaning “quality”), measures the percentage of correctly inspected items out of the total inspected items (including errors). Finally, the K measure, derived from the German word “Kontinuität” (meaning “continuity”), is determined by multiplying L and Q. K serves as the sole measure of attention and indicates the overall consistency of performance.

### Information-processing speed

A highly feasible measure of information-processing speed is the Trail Making Test (German: Zahlen-Verbindungs-Test; ZVT^31^). In this test, participants draw lines to connect, in order, numbers from 1 to 90, which are positioned randomly on a sheet of paper. The test comprises four forms, each containing numbers from 1 to 90 in various positions. Participants are instructed to complete each test form as quickly and accurately as possible. The average time taken to connect all numbers from 1 to 90 in the correct order across all four test forms was used as a measure.

### Sociodemographic data

All participants responded on the computer to sociodemographic questions, such as age, gender, eyesight, handedness, and mother tongue.

### Procedure

The experiment was carried out in individual settings in a soundproof booth (Studiobox, premium version) at the Teaching and Research Area Work and Engineering Psychology of the RWTH Aachen University. The participants were fully briefed on the components of the experiment and what was expected of them when they arrived at the lab. After signing the consent form, all participants underwent audiometry to verify that their hearing was within normal limits. They were then administered with the experiment. The complete test protocol was administered in a single test session. The experiment lasted about 90 minutes. Written instructions appeared on the screen for the dual-task paradigm and the aOSPAN or on a sheet form for the FAIR-2. For the ZVT, participants were instructed orally by the experimenter.

Participants started with the dual-task paradigm. At the start of the experiment, all participants took part in a practice session. Participants started with the primary listening task, consisting of one text spoken by two co-located conversing talkers. Before the participants could practice the vibrotactile secondary task, they were able to familiarize themselves with the tactile patterns and the corresponding button they had to press. For example, if the participants clicked the right arrow, the tactile patterns short-short and long-long were presented consecutively. The participants could do this as long as they liked. Then 20 practice trials of the pattern recognition task (vibrotactile secondary task) followed. After these two single tasks, each participant practiced dual-tasking, where the primary listening task, consisting of one text in the co-located condition, and the pattern recognition task had to be performed simultaneously. Participants were instructed at the start of the experiment that they should try to respond quickly and accurately to both tasks. After practicing single- and dual-tasking in the co-located condition, participants could listen to one text in the spatially separated condition to familiarize themselves with this condition. After the practice session, performance measures started. Here, the order of the three experimental tasks described below was balanced over participants to reduce the possibility of confounds due to presentation order^6^. The experimental tasks were (1) a single primary listening task, (2) a single vibrotactile secondary task, and (3) a dual-task – primary listening task and vibrotactile secondary task concurrently. In the single listening task, participants responded to two texts. The vibrotactile secondary task consisted of 40 trials. In the dual task, six texts (text order was randomized across participants) were presented for the primary listening task, while the number of trials of the vibrotactile secondary task was defined by the time of each text in the listening task. After each text, the corresponding questions were presented on the screen one after the other, and participants entered their responses via the notebook’s keyboard. These tasks (1, 3) were carried out either in the co-located condition or in the spatially separated condition (condition order was counterbalanced). A variable resting time was encouraged between these two conditions.

After participants completed the dual-task paradigm, they were administered the three psychometric tasks in a randomized order.

In the aOSPAN participants started with a practice session that was broken down into three sessions. First, participants practiced recalling sequences of consonants of set size two and three in ascending order (two repetitions each). After the recall, the computer provided feedback about the number of letters correctly recalled in the current set. Next, participants practiced 15 math operations. The participants were given instructions to solve the math operation and click on the mouse to proceed to the next screen. On the following screen, a digit was displayed, and participants were instructed to select either “correct” or “incorrect”. Each operation was followed by feedback on accuracy. The math practice was used to acquaint participants with the math portion of the task and to calculate each participant’s mean time required to solve the equation. This time (plus 2.5 *SD*) was used as a time limit for the math portion of the experimental session. In the last practice section, participants recalled sequences of letters and math problems, just as they would in the experimental session. Participants completed three practice trials, each of set size two. If their time to solve the math operation exceeded their individual time limit, the program proceeded automatically and recorded the attempt as an error. This method was implemented to prevent the participants from rehearsing the consonants rather than solving the math operations. After the practice session, the performance measures started. Participants underwent three repetitions of five set sizes (the order of set sizes is randomized), where they solved math problems and had to recall a sequence of letters.

At the start of the ZVT, participants were given a practice sheet containing randomly arranged numbers from 1 to 20. After the successful completion of the practice sheet, the performance measure started, and participants. The four test forms were undertaken consecutively, without any breaks in between them. The time taken by the participant to complete each test form was recorded using a stopwatch and initiated as the participant proceeded to the next form.

In the FAIR-2, each participant received a test form and was asked to read the standardized instructions printed on the form. After participants read the instructions, they practiced the task with one row of 20 stimuli. Upon successful completion of this practice row, participants were tasked with solving two pages with a time limit of three minutes per page.

### Statistical analysis

Data analysis was performed using R^37^. The dependent variables were the performance in the HTR task (binary variable: 1 for correct and 0 for incorrect responses), the performance in the secondary task (binary variable: 1 for correct vibrotactile pattern recognition and 0 for incorrect recognition), and the reaction times (RT) of correct trials in the secondary task (assessing the time from the appearance of a pattern to the participant’s response).

Generalized linear mixed-effect models (GLMMs) were used to model performance and RT data, employing the lme4 package^38^. GLMMs are more statistically powerful and better at capturing individual-level variability and dependencies between observations, which is why this approach was chosen over the traditional ANOVA^39,40^. RT data modeling was conducted only on correct responses, excluding incorrect responses or errors from the analysis. Before analyzing the RT data, RTs under 200 ms and outliers exceeding two standard deviations from the mean were identified and removed, following the procedure outlined in Berger and Kiefer^41^. This resulted in the exclusion of 5.5% of the RT data. Binomial distributions and logit link functions were used to model performance in the GLMMs, while a Gamma distribution and log link function were used to model RT.

Three different GLMMs were built to model the effect of *talkers’ audio condition* on performance in the primary task (HTR), as well as the performance and the RT in the secondary (vibrotactile pattern recognition) task. The final GLMM was selected through backward model selection. We compared the different models with Likelihood Ratio tests. When applicable, we conducted post hoc pairwise comparisons based on estimated marginal means (emmeans) using the emmeans package^42^.

For the GLMM modeling performance in the primary listening task, we considered *talkers’ audio conditions* (co-located vs. spatially separated), *number of tasks* (single listening task vs. dual-tasking), and the two-way interaction as fixed factors, and *information-processing speed* (mean RTs), *attention* (K score), and *working memory* (aOSPAN score) as covariates, and *age*, *question*, and *text* as random (intercept) factors. A random (intercept) factor refers to a variable that accounts for differences between groups that are not explained by the fixed effects in the model. In the final model analyzing performance in the primary listening task, all initially considered factors were included, except for the two-way interaction.

Participants completed the secondary (pattern recognition) task under three *task conditions*: (a) as a single task without the listening task, (b) as a secondary task in parallel with the listening task, with the talkers being either co-located or (c) spatially separated. The GLMMs modeled performance and RTs using the three *task conditions* (a)–(c) as fixed factors, and *information-processing speed* (mean RTs), *attention* (FAIR-2 K score), and *working memory* (aOSPAN score) as covariates, and *age* and *vibration type* as random (intercept) factors. In the final model analyzing performance and RTs in the secondary task, all initially considered factors were included, except for *vibration type*.

## Results

### Memory of conversational contents: performance in the primary listening task

**Fig. 2:**
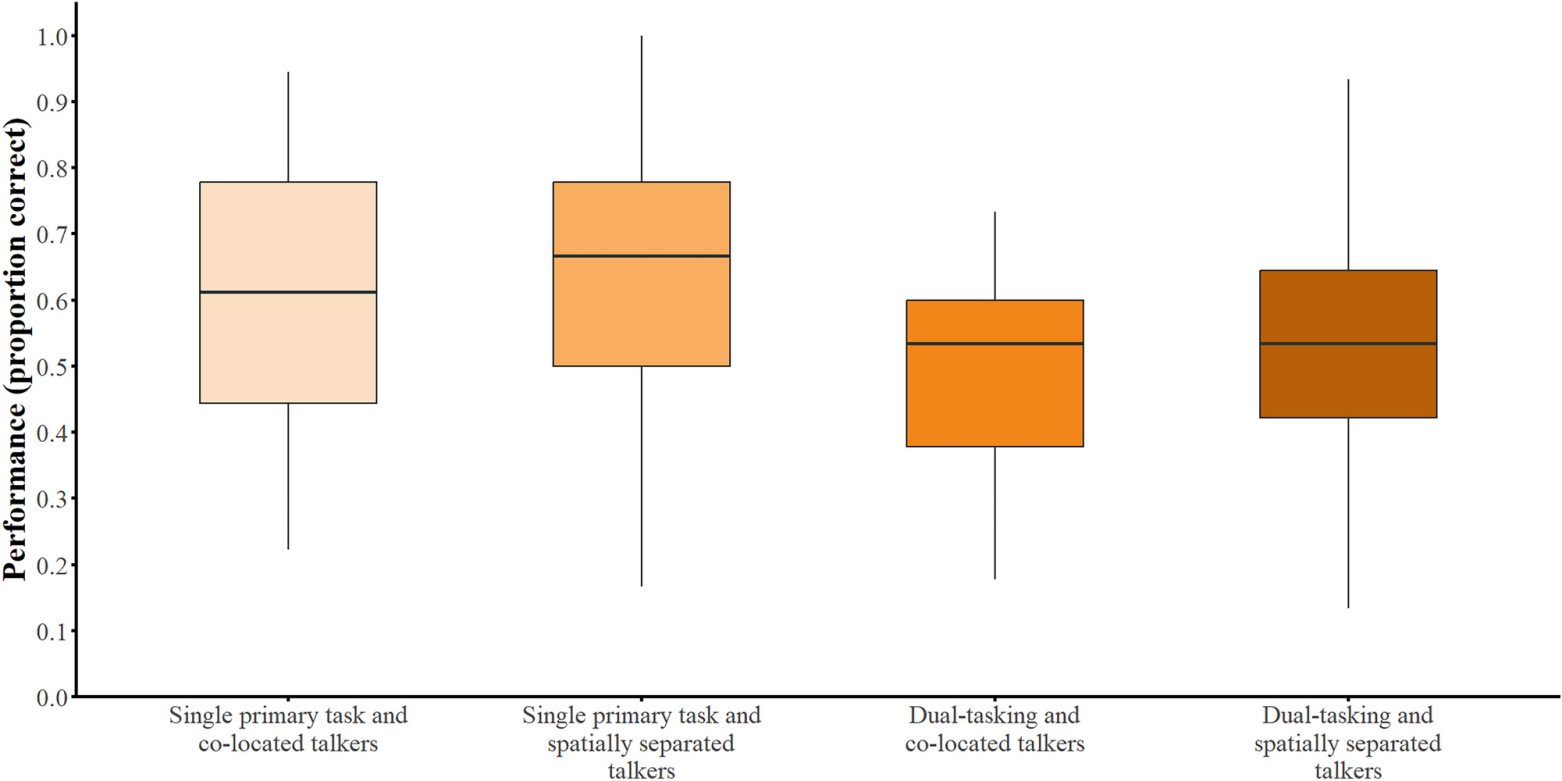
Performance in the primary listening task performance. Listening task performance as a function of *number of tasks* (single listening task vs. dual-tasking) and *talkers’ audio condition* (co-located vs. spatially separated). The boxplots show the data distributions for primary task performance (proportion correct). The lines inside the boxes represent the medians and the boxes represent the inter-quartile ranges.

First, the effect of *talkers’ audio condition* (co-located vs. spatially separated) and *number of tasks* (single listening task vs. dual-tasking) on memory for conversational content was investigated on performance in the primary listening task. The descriptive results are shown in Fig. 2.

The final GLMM model included *talker’s audio condition* and *number of tasks* as fixed effects, and *working memory, information-processing speed* and *attention* as covariates, and *age*, *question* and *text* as random (intercept) factors. Table 1 presents a summary of the final GLMM that modeled memory performance.

**Table 1:** Results from the final GLMM modeling performance in the HTR task as predicted by talkers’ audio condition.

Fixed effects and covariates	Estimate	*SE*	*t*	95% CI	*p*
Intercept	− 4.14	0.59	− 6.93	0.005, 0.05	< 0.001
Talkers’ audio condition					
Co-located	Reference				
Spatially separated	0.11	0.07	1.59	0.50, 0.69	0.11
Number of tasks					
Single listening task	Reference				
Dual-tasking	− 0.53	0.08	− 6.63	0.98, 1.29	< 0.001
Working memory	− 0.004	0.004	− 1.03	0.99, 1.00	0.30
Information-processing speed	0.04	0.004	7.88	1.03, 1.05	< 0.001
Attention	0.01	0.001	8.29	1.005, 1.008	< 0.001

Random effects	Variance	*SD*			
Age	0.37	0.61			
Question	0.21	0.46			
Text	0.07	0.26			

Number of observations: 3654; groups: age = 11, question = 9, text = 14. Confidence intervals calculated using the Wald method. Model equation: performance ~ block + direction + working memory + processing speed + attention + (1|age) + (1|question) + (1|text); family = binomial, link function = logit.

There was no significant effect of *talkers’ audio condition* on memory performance [χ^2^(1) = 2.52, *p* = 0.11], after accounting for *number of tasks*, *information-processing speed*, and *attention*. However, we did find a significant effect of *number of tasks* on memory performance [single- vs. dual-tasking; χ^2^(1) = 44.01, *p* < 0.001], after accounting for *talkers’ audio condition*, *information-processing speed*, and *attention*. A pairwise comparison indicated that memory performance was significantly better during single-tasking compared to dual-tasking (*z*-ratio = 6.64, *p* < 0.001).

### Listening effort: performance in the vibrotactile secondary task

**Fig. 3:**
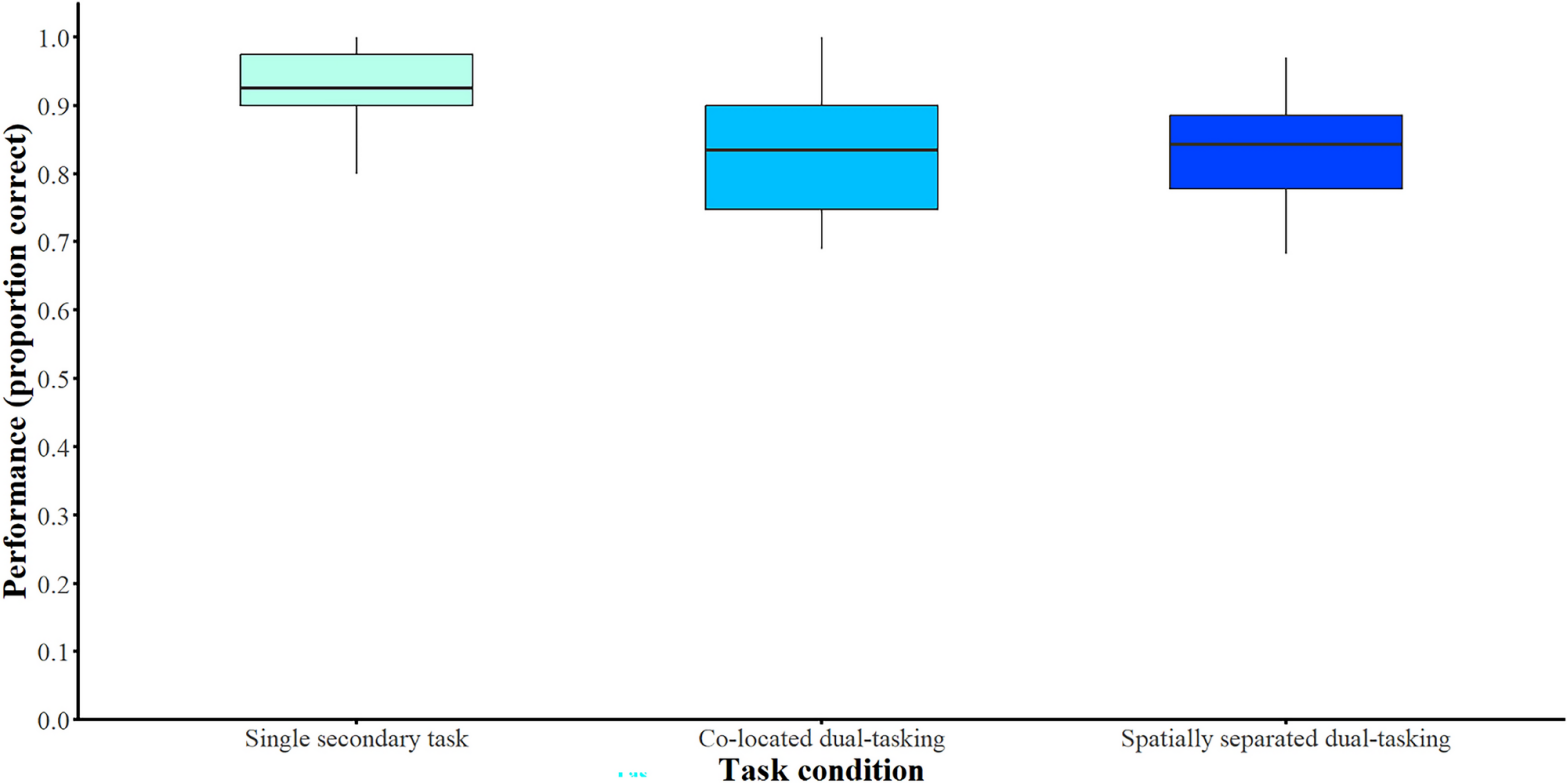
Performance in the secondary vibrotactile pattern recognition task. Secondary task performance as a function of task condition (single secondary task, co-located dual-tasking, spatially separated dual-tasking). The boxplots show the data distributions for secondary task performance (proportion correct). The lines inside the boxes represent the medians and the boxes represent the inter-quartile ranges.

**Fig. 4:**
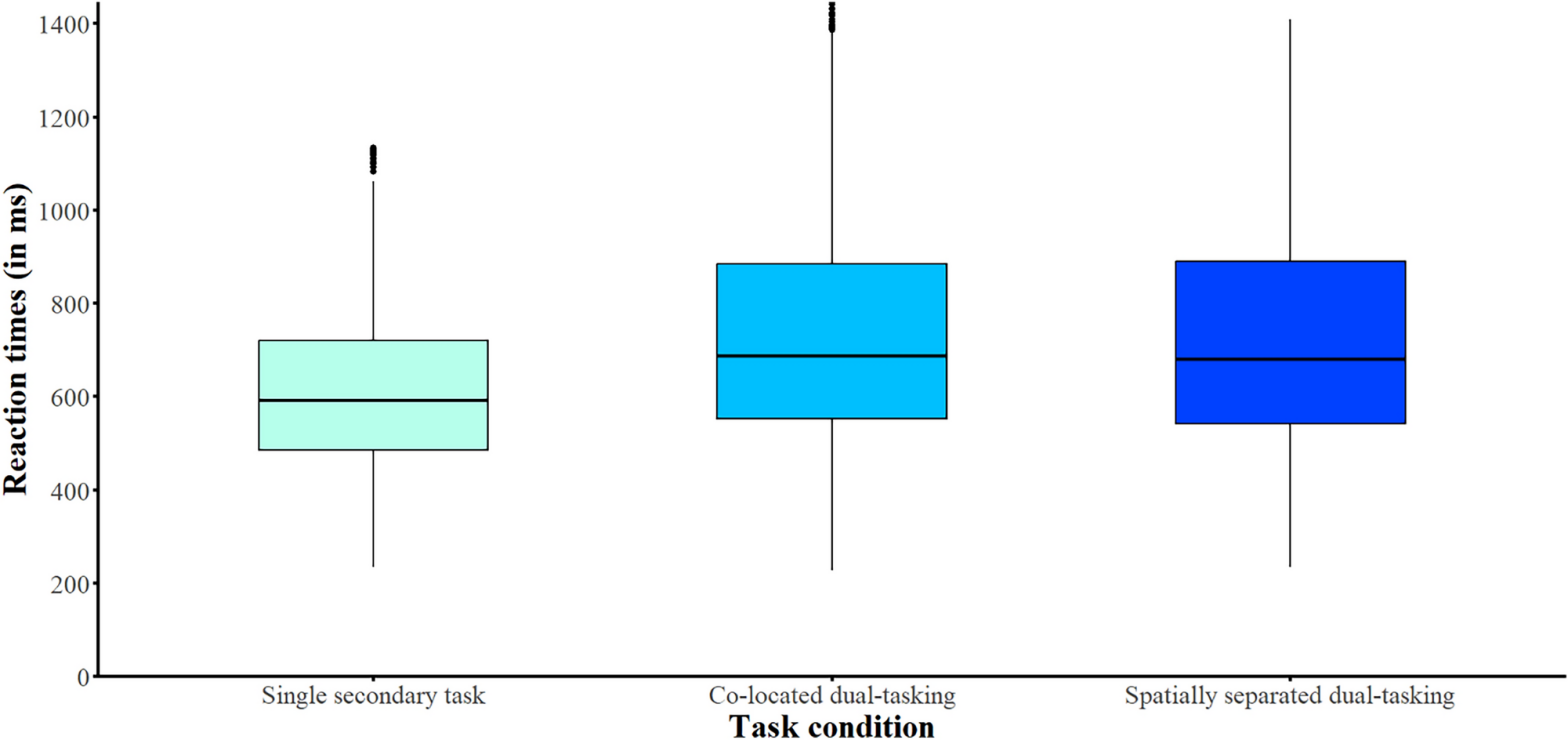
Reaction times in the secondary vibrotactile pattern recognition task. Secondary task reaction times as a function of task condition (single secondary task, co-located dual-tasking, spatially separated dual-tasking). The boxplots show the data distributions for secondary-task reaction times (in ms). The lines inside the boxes represent the medians and the boxes represent the inter-quartile ranges.

Next, the effect of *task condition* (a) single task without the listening task, b) secondary task in parallel with the listening task with the talkers being either co-located or c) spatially separated) on listening effort was assessed based on participants’ performance and RT in the vibrotactile secondary task. The descriptive results for performance and RT based on *task condition* are shown in Figs. 3 and 4, respectively.

The results of the GLMM analysis regarding performance in the secondary task are presented in Table 2. *Task condition* had a significant effect on performance in the vibrotactile secondary task [χ^2^(2) = 64.77, *p* < 0.001, after accounting for *working memory*, *processing speed*, and *attention*. A Bonferroni-corrected post hoc analysis revealed that performance in the secondary task was significantly better in the single-task condition in comparison to the dual-task condition with co-located talkers (*z*-ratio = 7.43, *p* < 0.001) and in comparison to the dual-task condition with spatially separated talkers (*z*-ratio = 7.90, *p* < 0.001). Nevertheless, the two *talkers’ audio conditions*, in which the primary listening task was presented simultaneously, did not influence secondary task performance (*z*-ratio = 0.79, *p* = 1).

**Table 2:** Results from the final GLMM modeling performance in the vibrotactile secondary task as predicted by task condition.

Fixed effects and covariates	Estimate	*SE*	*t*	95% CI	*p*
Intercept	1.86	0.49	3.76	2.44, 16.97	< 0.001
Task condition					
Single secondary task	Reference				
Dual-tasking with co-located talkers	− 0.89	0.12	− 7.43	0.32, 0.51	< 0.001
Dual-tasking with spatially separated talkers	− 0.95	0.12	− 7.90	− 0.30, 0.49	< 0.001
Working memory	− 0.01	0.003	− 2.21	0.98, 0.99	0.03
Information-processing speed	0.01	0.004	1.92	0.99, 1.02	0.06
Attention	0.001	0.0007	1.56	0.99, 1.00	0.11

Random effects	Variance	*SD*			
Age	0.17	0.41			

Number of observations: 7054; groups: age = 11. Confidence intervals calculated using the Wald method. Model equation: performance ~ task condition + working memory + processing speed + attention + (1|age); family = binomial, link function = logit.

The final GLMM results of the RTs in the secondary task are provided in Table 3. *Task condition* had a significant effect on RTs in the vibrotactile secondary task [χ^2^(2) = 255.06, *p* < 0.001]. This was observed when controlling for *working memory, processing speed,* and *attention*. Bonferroni-corrected post hoc analysis indicated that RTs in the secondary task were significantly higher during single-tasking compared to dual-task condition with co-located talkers (*z*-ratio = − 14.15, *p* < 0.001) and compared to dual-task condition with spatially separated talkers (*z*-ratio = − 13.41, *p* < 0.001). However, the two *talkers’ audio conditions*, in which the primary listening task was presented concurrently, did not affect the RT in the secondary task (*z*-ratio = 0.92, *p* = 1).

**Table 3:** Results from the final GLMM modeling reaction times in the vibrotactile secondary task as predicted by task condition.

Fixed effects and covariates	Estimate	*SE*	*t*	95% CI	*p*
Intercept	6.01	0.07	85.38	357.38, 471.08	< 0.001
Task condition					
Single secondary task	Reference				
Dual-tasking with co-located talkers	0.18	0.01	14.15	1.16, 1.22	< 0.001
Dual-tasking with spatially separated talkers	0.17	0.01	13.41	1.15, 1.21	< 0.001
Working memory	− 0.0001	0.0004	− 0.17	0.99, 1.00	0.87
Information-processing speed	0.002	0.001	4.41	1.00, 1.00	< 0.001
Attention	0.001	0.0001	8.38	1.00, 1.00	< 0.001

Random effects	Variance	*SD*			
Age	0.002	0.05			

Number of observations: 5620; groups: age = 11. Confidence intervals calculated using the Wald method. Model equation: reaction time ~ task condition + working memory + processing speed + attention + (1|age); family=Gamma, link function = log.

## Discussion

This study investigated how listeners’ working memory capacity, attention, and information-processing speed influence listening effort and memory for conversational content when listening to two-talker conversations in quiet settings. The two talkers’ audio signals were presented either at the same position (co-located) or spatially separated. The two talkers’ speech signals were presented in quiet listening settings, i.e. they were free of background noise and other sound sources.

Working memory, information-processing speed, and attention were used as covariates to account for the effects of auditory spatial cues on listening effort and memory of conversational content. Our findings underscore the associations between these cognitive functions and memory performance for running speech in a quiet listening setting. We found a significant relationship between memory of conversational content and cognitive functions such as attention and information-processing speed, emphasizing the importance of these covariates in listening to two-talker conversations. However, it is notable that working memory capacity did not have a significant effect on memory performance after accounting for other factors in the model. The findings suggest that attention and information-processing speed play a significant role in listening to running speech in quiet listening settings, while the present study could not verify such a role for working memory. In the applied listening task (HTR), attention is required to maintain focus on each talker in sequence and to shift between the female and male talkers, while information-processing speed allows the listener to keep up with the flow of the conversation, as the conversational exchanges are rapid and contain a variety of information (e.g. names, relationships, and ages). Because the two talkers were not speaking simultaneously and the listening environment was quiet, the overall cognitive load on working memory may have been relatively low. Once attention and information-processing speed are accounted for, the additional working memory capacity to hold and manipulate information may not significantly improve performance. At least this holds true for the applied listening task and the specific operationalization of working memory (aOSPAN^29^) in this study.

Furthermore, our examination of listening effort in quiet settings revealed connections with all three cognitive functions. Precisely, in the GLMM analysis for performance in the vibrotactile pattern recognition task, working memory emerged as a significant covariate. This indicates that participants’ (between-subjects) variations in working memory capacity were associated with differences in their task performance. On the other hand, attention and processing speed were found to be significant covariates in relation to reaction time rather than overall task performance. The vibrotactile pattern recognition task required participants to retain the first vibrotactile stimulus (whether it was long or short) in memory while awaiting and processing the second vibrotactile stimulus for comparison. This higher demand on working memory explains why individual differences in working memory capacity were significant in the secondary task. On the other hand, attention and information-processing speed played a significant role in managing the temporal dynamics of the task. In particular, differences in attentional focus and processing speed influenced how quickly participants focused and responded to stimuli during the task, rather than directly impacting their accuracy or success in recognizing the vibrotactile patterns. These findings highlight the distinct roles of these cognitive factors in the vibrotactile pattern recognition tasks, with working memory contributing to performance and attention and information-processing speed influencing reaction times.

Comparing our findings with those of Desjardins^23^, we find some similarities but also some differences. Both studies show the importance of processing speed and working memory in listening tasks. However, our study indicates that attention also plays a significant role in the present experiment. The differences in results between our study and Desjardins^23^ may be due to variations in the listening tasks used. Our study focused on memory of conversational content and listening effort during a two-talker conversation in a quiet setting. In contrast, Desjardins^23^ examined speech recognition of unrelated sentences and listening effort in noisy environments, which may emphasize different cognitive demands, e.g., higher working memory capacity. In addition, variations in the tasks used to measure individual cognitive functions may contribute to these different results. For instance, Desjardins^23^ measured attention with the Stroop test^43^, while we used the FAIR-2^30^. Overall, these differences highlight the importance of considering task-specific factors when interpreting findings on cognitive processing in listening tasks.

Our findings revealed no significant difference in memory performance between the audio conditions of the two talkers while considering the three person-specific cognitive functions. Precisely, participants did not recall more information when the talkers were spatially separated than when they were co-located, while accounting for working memory, information-processing speed, and attention. Additionally, we did not observe a reduction in listening effort for spatially separated talkers, which contrasts with the findings of Fintor et al.^3^, who reported a performance benefit in secondary tasks when the talkers were spatially separated. This discrepancy might stem from differences in the primary listening task. While Fintor et al.^3^ also administered the Heard Text Recall task^34^ as a primary listening task, they used speech stimuli from a single professional female talker whose voice was pitch-shifted to mimic a male voice. This preserved uniformity in speaking styles, prosody, and speech pace. In contrast, our study utilized distinct female and male talkers. Despite efforts to maintain normal intonation, variations in other vocal characteristics, such as speech pace, might have occurred. These differences could have facilitated the perception of the two talkers as separate entities in the co-located condition, so that their spatial separation did not further release a reduction in listening effort. Further investigation is warranted to explore how various auditory cues, including voice features, speaking styles, and spatial positioning, influence listening effort and to elucidate the reasons behind the divergence between our and Fintor et al.’s^3^ findings.

Our findings also suggest that the availability of auditory spatial cues does not always alleviate listening effort compared to simpler reproduction methods typically employed in cognitive-psychological experiments. Referring to the ELU model^2^, our results support the notion that spatially separated and co-located talkers did not elicit different processing routes. However, this might only apply to quiet settings and the primary listening task (HTR^3,34^) used in this study.

Previous research has largely focused on understanding the mechanisms of hearing and listening using simple cognitive tasks under adverse listening conditions. However, it may be beneficial for auditory research to incorporate more ecologically valid cognitive tasks, such as memory of running speech, in quiet listening settings and further explore the relevance of person-specific cognitive capacities and capabilities. Individuals naturally differ in their cognitive resources, including working memory capacity, attention, and information-processing speed. Accounting for these variations can provide valuable insights into how individual cognitive functions influence memory of conversational content and listening effort in quiet listening settings.

## Supplementary Information

Supplementary Information 1.

Supplementary Information 2.

## Data Availability

All data generated or analyzed during this study are included in this published article (and its Supplementary Information files).
